# An understanding of falling bodies across schooling and experience based on the conceptual prevalence framework

**DOI:** 10.1186/s43031-023-00075-4

**Published:** 2023-06-01

**Authors:** Patrice Potvin, Pierre Chastenay, François Thibault, Martin Riopel, Emmanuel Ahr, Lorie-Marlène Brault Foisy

**Affiliations:** Département de Didactique, Université du Québec à Montréal, C.P. 8888 Succ. Centre-Ville, Montréal, Québec H3C 3P8 Canada

**Keywords:** Misconceptions, Physics, Falling bodies, Coexistence, Response times, Conceptual attractors

## Abstract

In this article, we describe a study conducted online with 953 participants of varying levels of education and, when applicable, science/physics teaching experience. These participants were asked to solve a cognitive task in which many different pairs of objects were presented and to identify which, if any, would touch the ground first when dropped (in atmospheric or non-atmospheric environments). Recorded accuracies and response times allowed us to conduct an analysis based on the conceptual prevalence framework, which posits that the coexistence of conceptual and/or *misconceptual* resources can produce interference in response production. The results show that the influence of some of them decreases or, more surprisingly, increases with training. In fact, secondary and college physics teachers seem to cultivate some of them, and most likely have contributed to their spread. The implications for teaching and research are discussed.

## Introduction

### The considerable challenge of teaching and learning about falling bodies

Physics teachers know that learning mechanics is not a straightforward, trivial, or easy process. Even though students are constantly witnessing manifestations of forces in their immediate environment, and even though they have heard of and used the concept of force, this familiarity does not necessarily seem like an advantage. In fact, in a physics course, applying “common sense” (i.e. superficially or locally useful, while nevertheless intuitive knowledge [Sherin, 2006]) about forces and motion often leads to failure: “A person may possess a perceptual appreciation of the natural dynamics of physical events, yet be unable to draw upon this knowledge when asked to conceptualize an event’s outcome in a representational context” (Kaiser et al., 1985, p. 539). Even though research regularly records decreases of misconceptions with schooling (Bayraktar, 2009, p. 273), a comprehensive pedagogical solution that could considerably facilitate teaching still appears far from reach. After submitting over 6000 students to the *Force Concept Inventory* (FCI), a classic multiple choice and widely validated test (Halloun & Hestenes, 1985) that sets traps for participants through conceptually appealing distractors, Hake (1998) concluded that traditional courses fail to convey much basic conceptual understanding of Newtonian mechanics to the average student. The FCI is a typical example of the questionnaires that are often used in the science education research subfield called “conceptual change,” which central idea is that students are adherents to conceptual frameworks that are often different from that found in students’ textbooks and taught by their instructors. Often called “misconceptions” when in partial or complete contradiction with scientifically validated knowledge, these honest but flawed frameworks and resources can explain recurrent errors, especially in fields like mechanics, where learners have grown and evolved in an environment that somehow keeps general invariants hidden because of its specificity.

### Misconceptions that simplified falling bodies problems often reveal

We chose to limit our study to falling bodies problems, which are among the most commonly taught and most studied in physics education research. Somehow emblematic of the counterintuitive connotation of mechanics (for example, the very first question of the Force Concept Inventory -FCI (Halloun & Hestenes, 1985) is about falling bodies), they are also sometimes addressed in famous public education initiatives (see this popular short video). We thus chose to exclude parabolic falling bodies problems that concentrate on lateral movements (Kaiser et al., 1985), quantitative relations between fall, mass and height (Vicovaro, 2014) and terminal velocity (Ferreira et al., 2017). Since atmospheric falls with higher acceleration also necessarily show higher terminal velocity (*Ibid*.), merely concentrating on the duration of the fall (from equal heights) is an acceptable reduction of falling bodies problem while remaining pedagogically informative about the forces one should consider.

Simple, reductive problems involving two different objects, such as the classic “Galileo from the Tower of Pisa”, also allow for the avoidance of computation or cognitive overload and have been shown to reveal misconceptions. The literature also suggests that simple comparisons of what happens in the context of different environments (*e.g*. atmospheric Earth and non-atmospheric Moon) can be pedagogically informative (Cahyadi & Butler, 2004).

Not surprisingly, the most widely studied and addressed misconception in simple falling-body problems establishes a close, causal relationship between mass and velocity of fall, so that participants often respond that heavier objects will tend to touch the ground first. The existence of this misconception, which is often documented in younger children with less exposure to physics courses (Van Hise, 1988), has been studied very early in the history of conceptual change (Champagne et al., 1980; Gunstone & Watts, 1985; Halloun & Hestenes, 1985; Sequeira & Leite, 1991; Whitaker, 1983) and has been shown to be omnipresent in almost all vertical motion justifications (Hast & Howe, 2012). It appears to be frequently invoked from ages five (*Ibid*.), seven (Van Hise, 1988), eleven (Song et al., 1996) and even throughout adulthood.

However, the justifications that participants of different ages or training levels use to support their adherence to “mass speed” are not always equivalent. While some invoke simple “logic” or their “experienced observations” (junior high) (Champagne et al., 1980), more knowledgeable students (undergraduates) invoke “more force” (Syuhendri et al., 2019), or “more gravitational pull” exerted on the heavier objects (Whitaker, 1983). Sequeira and Leite (1991) provided a delightful example of a graduate student who even “knowledgeably” invoked the law of universal attraction (F_g_ = Gm_1_m_2_ /r^2^) to justify that objects having greater mass will necessarily be “more rapidly attracted.” In this particular study, more than 50% of fourth-year university physics students formulated justifications exclusively based on weight or mass. The origin of such a conception could be merely experiential: Vicovaro hypothesized that “the mass-speed belief occurs because people extend their perceptual-motor experience of holding objects in hand to free fall” (Vicovaro, 2014, p. 467). She then confirmed this origin through an astute experiment comparing perceptual assessments of falling objects that were either based on mere *knowledge* about weight (communicated information), or on “felt” weights. Consistent with Kavanagh and Sneider (2007), Gunstone and Watts (1985) argued that such a student's idea is so stubbornly held that it affects the interpretation of his or her observation. They cited a case in which a student, identified by his teacher as academically gifted, claimed to have observed heavier objects hitting the ground first in the past. Chinn and Malhotra (2002) have also confirmed such conceptually driven observational biases (see their Table 2, p. 331).

But observations that heavier objects fall faster are very difficult to contradict, since they can indeed be made: a pebble will hit the ground before a feather, and a beach ball filled with water will also hit the ground before a similar one filled with air (even though they have the same volume). The speed-mass misconception therefore does not always lead to errors in prediction. It clearly appears as a misconception in the lunar context (free fall without atmospheric drag), but in Earth’s atmosphere (drag-effect), mass and shape can never be completely ignored, except right at the beginning of the fall, when drag is null and the fall conforms to free fall (accelerated at 9.8 m/s per second on Earth). But immediately after this point in time, drag appears and creates a force upward (*i.e*., a decelerating force) that is proportional to the density of the displaced fluid (air) [ρ], the speed [v] of the falling object relative to this fluid (squared), the cross-sectional area of the falling object [A_c_] (as seen from directly below), and the drag coefficient (a dimensionless quantity that depends, among other things, on the shape and orientation of the object; for a sphere, this value is around C_d_ = 0.47). From the equation, one finds that the deceleration (*i.e*., negative acceleration) due to drag on a falling object with air resistance is proportional to the ratio A_c_/m (cross section/mass). So, when the problems assigned to students compare falling objects of different masses but of the same shape (constant A_c_), in the same environment (constant air density) and from the same height, the rate at which an object falls (*i.e.,* its acceleration) diminishes with increasing A_c_/m, and consequently, objects with “the same value for (A_c_/m) fall at the same rate” (Ferreira et al., 2017, p. 4). Thus, “In the case where both objects have the same A_c_, this boils down to something that closely resembles the alternative conception students report: the more massive object, having a lower value for A_c_/m, will fall more rapidly” (*Ibid*.), since the deceleration due to atmospheric drag will be lower. Another way of putting it would be to imagine spheres of uniform density that differ by their radius: while mass grows proportionally to the *third power* of the radius (m = 4/3πr^3^*density), drag is only proportional to the *second power* of the radius (because A_c_ = πr^2^). Thus, a small, rather spherical pebble detached from a larger, more-or-less spherical boulder in free fall will necessarily hit the ground *after* the large boulder (in a dragging atmosphere), since the boulder’s A_c_/m will be much smaller than the pebble’s (*i.e*., its deceleration will be less). However, depending on the difference in Ac/m ratios between the objects being compared, differences in fall durations may be more or less readily apparent and may require greater fall heights (and thus durations) to become apparent (Ferreira et al., 2017). For example, the difference in fall duration between a feather and a pebble can easily be observed with the naked eye on a fall of less than a metre high, since the feather’s A_c_/m is much larger than the pebble’s. On the other hand, the difference between a plasticine ball and another one 20% smaller might necessitate a fall from a height of many tens of metres to show a difference. Ferreira et al., (2017, p. 7) concluded that only “if the A_c_/m ratio of a ball is more than the critical ratio for a certain height […] the effect of air resistance can be ignored.”

Thus, experiments and observations carried out in an approximate way can, in some cases, reinforce the “mass-velocity” conception, while in other cases they can reinforce the adherence to Galileo's principle of free fall. This considerable difficulty can lead to all sorts of errors that we can find in students’ verbalizations, as well as in many websites, teaching initiatives (even some of the “best” ones), and even research designs. For example, in his 1983 study, Whitaker (1983, p. 356) proposed a diagnostic test that required participants to respond to this question: “A 5-pound ball and a 50-pound ball are dropped from the roof of a building. Each ball has the same size, that is, it has the same diameter. The 50-pound ball will hit the ground: (A) Sooner than the 5-pound ball; (B) Later that the 5-pound ball; or (C) At the same time as the 5-pound ball. Surprisingly, Whitaker identified the answer “C” as the correct one (1983, p. 353), instead of “A”. Chinn and Malhotra (2002), in their famous study about “Children’s Responses to Anomalous Scientific Data,” also make a similar error, as they affirm that.

*“the correct interpretation in the rock-dropping experiment is that the rocks [a heavy* + *a lighter one] fell at the same speed; incorrect interpretations assert that one of the rocks fell faster than the other […] indicating whether their responses are conventionally correct from the point of view of accepted scientific theory.” (p. 237).*

Such mistakes, surprisingly made by authorities, are indicative of a somewhat abusive extension of Galileo’s principle to all contexts, with and without drag. It is reasonable to assume that such a misconception comes from honest, while possibly imprudent, teaching initiatives: “After encountering some formal instruction at the secondary or high school level, students are able to say that both balls will hit the ground together; however, this is not always supported by correct explanations or reasoning. Some may have memorized a statement seen in a textbook or heard it from a teacher” (Cahyadi & Butler, 2004, p. 572). In a few cases, a clear misunderstanding of Galileo’s principle leads participants to believe that all falling objects in a gravity field (say on Earth) are submitted to the same exact amount of force. This belief even leads some of them to a conclusion that directly opposes the mass-speed conception: “The Earth applies the same force to all bodies thus a lighter one gets more acceleration” (Song et al., 1996, p. 169).

In any case, Galileo’s principle remains applicable per se only in the context of perfect vacuums, like in problems for which students are told to “ignore resistance.” Commonly presented as “in direct conflict” with the “mass-speed” conception (Ferreira et al., 2017, p. 2), we believe that such an opposition should never be presented as absolute or exclusive. But Lehavi and Galili (2009) observed in students that “The most prevalent confusion was between the status of Galileo’s law as an empirically based statement that *replaced* the Aristotelian *heavier bodies fall faster*” (p. 421). Records of such a tension between mass-speed and Galileo’s principle have been long known and qualitatively reported [e.g., “Any object no matter the mass will fall at 32 ft/s^2^” (Whitaker, 1983, p. 353)]. Even if students are eventually capable of commonly producing correct answers involving Galileo’s principle and can “repeat what they have been told,” they still sometimes confess that, in fact, they “never really believed it.” (Arons & Redish, 1997). Champagne et al. (1980) also provide a good example of hesitations in one participant, who said: “there is something in the back of my mind which says that an object falls at a constant rate”. (p. 29–30).

This may be why so many students [sometimes as many as 50%, according to Sequeira and Leite (1991)] choose to completely ignore the influence of air resistance on differently shaped objects, even when the falls being considered clearly occur in the air and the objects being compared clearly have different shapes (Cahyadi & Butler, 2004, p. 573). Ferreira et al. (2017) suggested that the examples commonly used in physics classes may help to reinforce such a drag-ignoring conception:

*“[…] a parachutist experiencing an increase in drag when he opens his parachute. […] For example, in comparing the motion of a leaf and a nut falling from the same tree and the nut landing first, the difference in motion is more often explained in terms of the bigger mass of the nut than in terms of the bigger cross-sectional area of the leaf.” (pp. 7–8)*

As in this “parachute” example, many students who adhere to Galileo’s principle may be confused by certain cases, such as the feather-pebble problem or the two beach balls (filled with air/water). For this reason, many of them may invoke some sort of “parachute” effect, where, for example, larger objects with the same mass experience more drag and reach the ground later. Cahyadi and Butler (2004, p. 575) noticed that “It turns out to be easier for the students to imagine the situation with balls of different density. This understanding may be acquired from the more obvious effect in everyday life of air resistance on different-sized objects, as compared to the one involving different mass objects. For example, larger (less dense) objects tend to move more slowly in the air than smaller ones.” Density appears to be a more acceptable (and thus more predictive) approximation of the Ac/m ratio (multiplicative inverse). But if we may hypothesize that some students resort to “impressionist” density estimations, few of them seem to explicitly invoke them to justify their answers. Some participants in reviewed studies also sometimes invoke “smoothness” (slickness) as an important variable, however never very strongly. Indeed, in physics manuals, smoothness is currently “methodologically” dismissed right from the beginning, in the initial formulations (“two objects […] with same smoothness…”).

The cases where volumes are different, but masses are similar can be solved intuitively through experience, but the cases where volumes are the same, but masses are different seem much more problematic, since mass, unlike volume, is not a readily “visible” property. Cahyadi and Butler (2004, p. 569) have observed that “the [undergraduate] students understood the impact of air resistance on the object’s size better than the impact of air resistance on objects of the same size but different mass.” Students often reason that “the effect of air friction would be the same due to the similar size of the balls” (*Ibid*. p. 572), leading many yet-to-be competent physics students (and even some teachers) to see the opportunity to apply Galileo’s principle and ignore their different weights, even though they should not because, as we have shown earlier, “the mass also influences the time taken to hit the ground when there is air resistance” (*Ibid*.). It appears a bit paradoxical that in such cases the “mass-speed” conception—frequently seen in younger, pre-physics-training students—appears, in the end, as not being a misconception at all, and instead participates in the production of correct answers.

### Students’ conceptual dynamics and its conceptual attractors

The previous analysis suggests that to better understand students' conceptual dynamics about simplified falling body problems, it may be preferable to avoid looking for the mere presence or absence, or appearance and disappearance, of certain monolithically described misconceptions. Our analysis of the previous literature suggests that participants' conceptual dynamics show hesitations, tensions, high contextuality, and fluidity.

As Brown and Hammer (2008) cleverly described, they are more or less drawn to “conceptual attractors”, which are understood as conceptual resources learners tend to invoke and use in flexible ways to produce satisfyingly plausible answers, while not necessarily stiffly committing to them. In this exact line of thought, Cahyadi & Butler argued that “a misunderstanding of the physics is often not wrong but rather a misapplication of the correct conception or a misunderstanding of to how to use other elements in students’ resource banks.” (2004, pp. 579–580) This idea that conceptual elements are available to students in a “resource bank” appears to be fundamental to a “complex systems perspective” (Brown & Hammer, 2008). From the analysis of the misconception literature presented above, we have extracted a list of relevant “conceptual attractors” that are thought to influence students” responses to falling body problems. These attractors have been chosen because of the attention they have received in the past. We have attached a label to each of these attractors that will be used throughout this study:

• VOLUME is the tendency to believe that larger (or wider) objects will necessarily fall faster. This *misconceptual* resource is usually recorded with very young children (Song et al., 1996).
• MASS is the inclination to believe that heavier objects necessarily fall faster, regardless of other characteristics or circumstances (Champagne et al., 1980).
• SIMULTANEOUS is the inclination, possibly based on partial understanding of physics training (Galileo), that all considered objects in a problem would reach the ground simultaneously, regardless of all other characteristics or circumstances (Cahyadi & Butler, 2004).
• PARACHUTE (sometimes called the “drag effect” (Ferreira et al., 2017)) is the tendency to believe that larger objects (greater surface area) show an increased tendency to be slowed down, in all contexts. Participants rarely invoke this attractor on its own, most likely because drag alone cannot explain falls. However, sometimes they do. This attractor can also be invoked to explain exceptions to SIMULTANEOUS, such as why a feather does not fall as fast as a pebble.
• SMOOTH is the tendency to believe that slicker or smoother items will fall faster than ones that show rougher surfaces. This conceptual resource is rarely used in isolation, and is usually subordinated to other considerations, or plainly ignored (Ferreira et al., 2017). Our task nevertheless allows testing it for comprehensiveness.
• GALILEO is the inclination of applying Galileo’s principle in its correct, non-atmospheric context. It is a more discriminate application of SIMULTANEOUS (Lehavi and Galili (2009)).
• MASS-DRAG is the expert’s inclination to simultaneously integrate the influence of mass and drag into atmospheric falling bodies problems. Since our idealized task is qualitative by nature and requires participants to answer rapidly, we believe that experts could most likely proceed by impressionist approximations of density or of A_c_/m ratios (Ferreira et al., 2017).

In the normative context of science education, we will here consider that GALILEO and MASS-DRAG are scientifically preferable to the other identified attractors, in non-atmospheric and atmospheric contexts, respectively.

### The conceptual prevalence framework

There are many perspectives that have attempted to describe the process by which conceptual understanding of scientific concepts can improve despite the initial presence of misconceptions. In the more classical streams of the 1980s (Nussbaum & Novick, 1982; Posner et al., 1982), conceptual change was viewed as a process of substitution (diSessa, 2006). In this perspective, cognitive conflict was central, understood as a mental state that triggers and motivates substitution. A decade later, conceptual change was rather seen as a transformative process, constrained by an ecology of big and small cognitive resources, like intuitive rules (Stavy et al., 2006), core intuitions (Brown, 1993), p-prims (diSessa, 1993), or ontological frameworks (Chi, 1992; Vosniadou, 1994). In the first decade of this century, the conceptual change research field recorded more interest in sociocultural perspectives and practices (Kelly & Green, 1998; Mason, 2007), which were judged to better simulate the authentic functioning of scientific communities, making conceptual change a process of collective negotiation or mediation that was based on facts and triggered by sociocognitive conflicts (Perret-Clermont, 1996).

However, since the beginning of the 2010s, the already suspected hypothesis that scientific misconceptions are neither erased nor transformed during conceptual changes has been repeatedly confirmed, through mental chronometry (Potvin et al., 2015), functional magnetic resonance imagery (Brault Foisy et al., 2015; Masson et al., 2014) and electroencephalographic methods (Skelling-Desmeules et al., 2021; Zhu et al., 2019). The persistence of misconceptions has even been confirmed with secondary science teachers (Potvin et al., 2015), PhDs in physics (Allaire-Duquette et al., 2021) and university chemistry professors (Potvin et al., 2020).

**Fig. 1:**
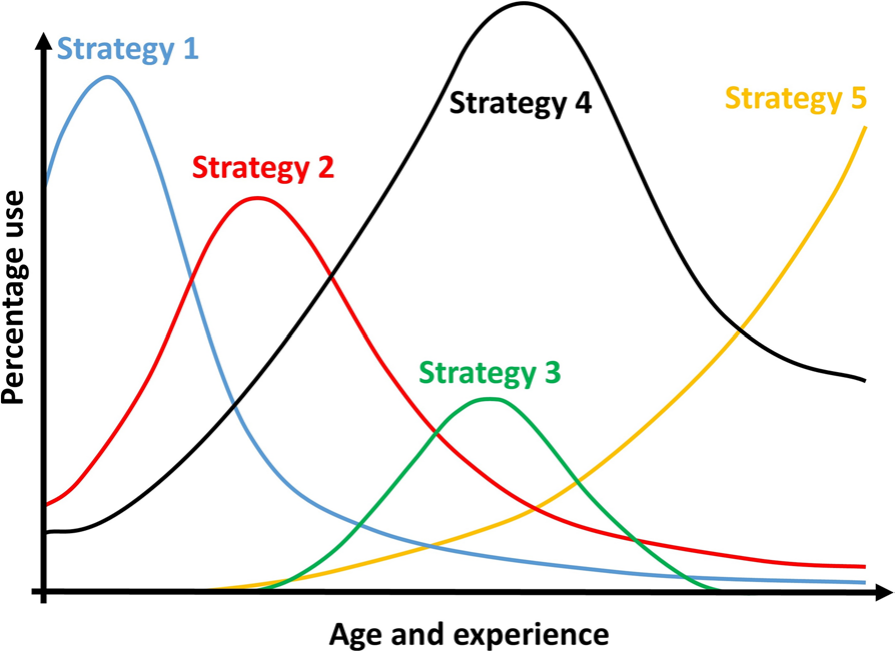
Recomposition of the “overlapping waves model”, by Siegler (1996)

Thus, recent models of conceptual change have proposed to accept the idea that different and sometimes contradictory conceptions (or conceptual resources) can coexist in a learner’s mind (Ganea et al., 2020), or can be mobilized unequally depending on the context, or on the level of competence (age and experience). During problem resolutions or prediction requests, these conceptual resources (*i.e*., attractors) are put in competition, and eventually, some of them—or some combinations of them—eventually prevail. Such a pluralist view (Bélanger & Potvin, 2022, 2022) supports the perspective of the “overlapping waves model” (Siegler, 1996), in which preference for conceptual solutions can vary with context, but also over age and experience (van der Ven et al., 2012; Wang & Wang, 2015) (Fig 1).

In﻿ the perspective that we have adopted, the *adherence* to all coexisting conceptual attractors may vary with experience and context, but *prevailing* conceptions never completely replace their competitors. Experienced learners can however suppress or inhibit them (Shtulman & Valcarcel, 2012). Non-prevailing conceptual resources however may remain tempting, and may consciously or unconsciously participate in internal deliberations, in which case they produce recordable *interference*. Here we used the central concepts of (1) *adherence*, (2) *prevalence* and (3) *interference*, for which we provide the following definitions (Potvin & Cyr, 2017, p. 1125):

- *1. Adherence* “can be defined as the level of perceived cognitive utility that a person attributes to a conception (or a misconception) in a specific context of performance or response in relation to other possible competing conceptions. It can be considered higher when a person succeeds more often at congruent/intuitive tasks (in which there is no conceptual distractor and are aligned with the tested conception) and fails more often at incongruent/counterintuitive ones (in which conceptual distractors could derail responses consistent with the concept being tested). […]”;
- *2. Prevalence* “would be the special level of adherence of a (mis-) conception when it is superior to the ones of all other presumably existing conceptions in a certain context. Most of the time, we are only conscious of our prevalent conceptions. And when learners must defend their choices, justifications that support prevalent conceptions usually appear first and are often expressed with strength and sometimes with a feeling of certainty […].” [For example, if a learner's misconception is prevalent, such as “magnets attract metal”, he/she might consistently and without hesitation state that magnets attract aluminum when prompted.]; and
- *3. Interference* “can […] be defined as the distracting effect of a non-prevalent conception on a particular performance or response. The greater this interference, the longer it will take to produce correct performances or answers.” [For example, if a learner is asked, “Are trees living things?” he/she may hesitate longer if he/she has more than one credible but incompatible idea to rely on (i.e., “all living things are mobile” and “all living things reproduce”)].

These definitions will be used to frame further efforts in measuring the perceived cognitive utility (through accuracies [prevalence] and response times [interference]) of learners with each of the seven identified conceptual attractors. Recent studies postulating the coexistence of multiple misconceptions have thus used mental chronometry—in addition to measures of accuracy—to infer the presence of interfering misconceptions. Using tasks that are typically very simple and that may or may not involve misconceptions, researchers postulate that longer cognitive processing times for correctly answered incongruent stimuli (in which a frequent misconception could threaten accuracy) are valid indicators of interference, and thus of the presence of such a misconception. Of course, such an interference effect can only be inferred if there is no other plausible source of interference and if the incongruent and congruent stimuli are equivalent in complexity.

### Statement of the problem and research question

The literature examining the misconceptions that prevail or interfere in falling bodies problems is extensive. However, it remains fragmented and few research initiatives have attempted to provide descriptions of conceptual evolutions over long periods. We thus believe that we need and extended portrait across the lifespan of the conceptual challenges that teaching about falling bodies poses to educators. The research question thus becomes: *What is the evolution of the prevalence of- and the interference by- conceptual attractors in falling bodies problems, as a function of schooling and of teaching at different levels?* We formulate the hypotheses that the perceived cognitive utility of *misconceptual* resources will decrease with schooling (and teaching) and that the adherence to each scientific conception will increase. This analysis is an original contribution because it provides, with one single task, not only an account of correct or incorrect answers, but also of the adherence (and “virulence”) to many conceptual attractors (pluralist account). It is also original because it provides for the first time (to our knowledge) an examination of the use of *misconceptual* resources in falling bodies problems that happen with- as well as without- atmospheric contexts, despite the important number of studies about such misconceptions.

The investigation has been facilitated as physics (mechanics) has been taught and learned at the same levels in the province of Québec (Canada) since at least the 1960s. Indeed, in the last 63 years, mechanics and falling bodies problems have been taught almost exclusively in secondary school as an optional course (5^th^ year—around age 16), at the college level for “natural sciences” students only (pre-university college [*CEGEP*]—around ages 17–19); and at the university level (for physics students as well as for preservice high school science teachers—around ages 20–24). Each of these courses had the precedent as prerequisite. Thus, all students who succeeded in at least one mechanics class at university had also succeeded beforehand in college and, previously, in secondary-level mechanics courses.

## Methodology

### Participants

Participants were 953 individuals of all ages and levels of education. Recruitment was conducted through formal institutions (elementary and secondary schools and school boards, colleges, universities), and informal means (publicity on social media and in teachers’ groups and pages). Permission to communicate with students during their courses was obtained from participating school boards (*n* = 4), private schools (*n* = 9) and colleges (*n* = 5). The process of recruiting for and conducting the experiment lasted approximately six months (November 2020 to April 2021), and recruitment was continuously adapted to ultimately obtain enough participants in each age group/training category. Each participant was only tested once to ensure spontaneity, so the design of the study is transversal.

### Materials

We designed a cognitive task on the *PsychoPy*™ (v.2020.2.8) software and made it available on the *Pavlovia.org* online platform. This task could only be run on computers with a keyboard (to record response times uniformly) and took less than 7 min to complete.

**Fig. 2:**
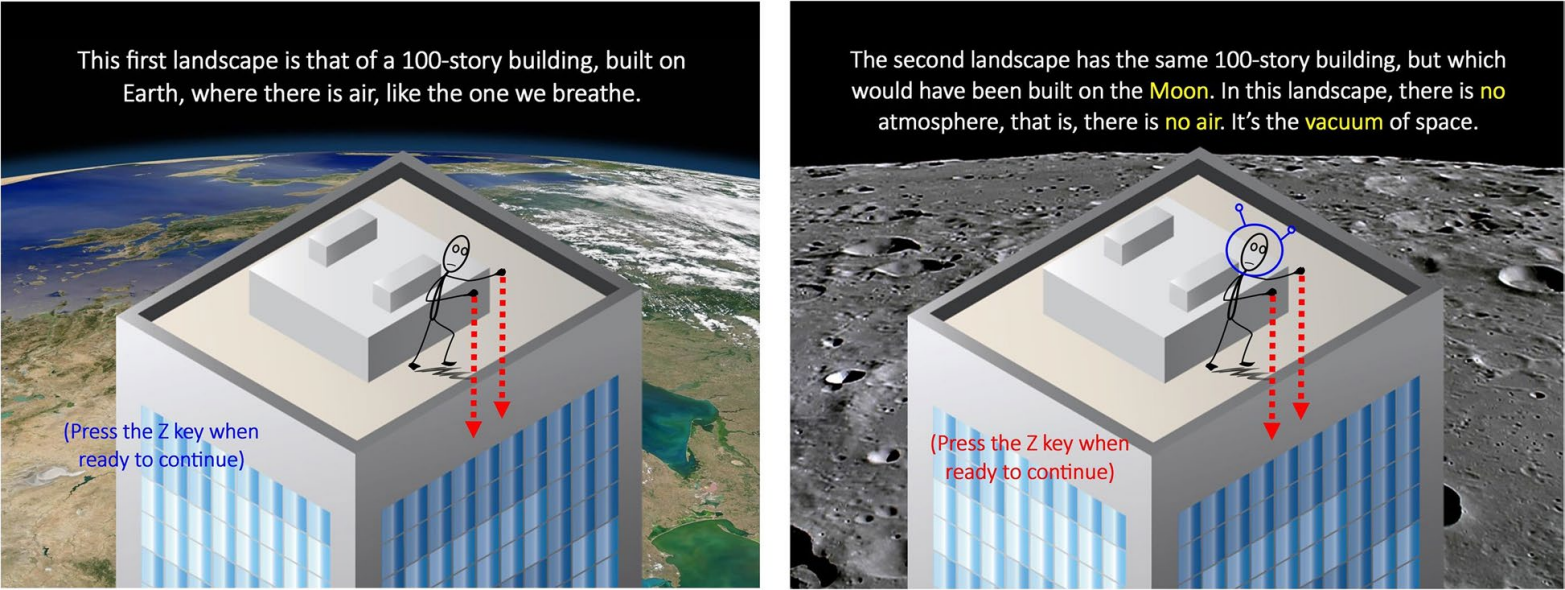
A few important slides from the instructions

After answering simple questions about consent, level of training and/or teaching mechanics, participants read the description of the problems to be solved (See Fig. 2 for a few slides). In a nutshell:

*A person simultaneously releases two objects from the roof of a 100-story building, to see which one will hit the ground first. […] This can happen on Earth (where there is air, like the air we breathe) or on the Moon, where there is no air. […] You will witness these experiments and try to predict, but never witness, the outcome (the object that appears on your left will hit the ground first [hit the “left arrow” key], the object that appears on your right will hit the ground first [“right arrow” key]; or both will hit the ground simultaneously [“down arrow” key]).*

**Fig. 3:**
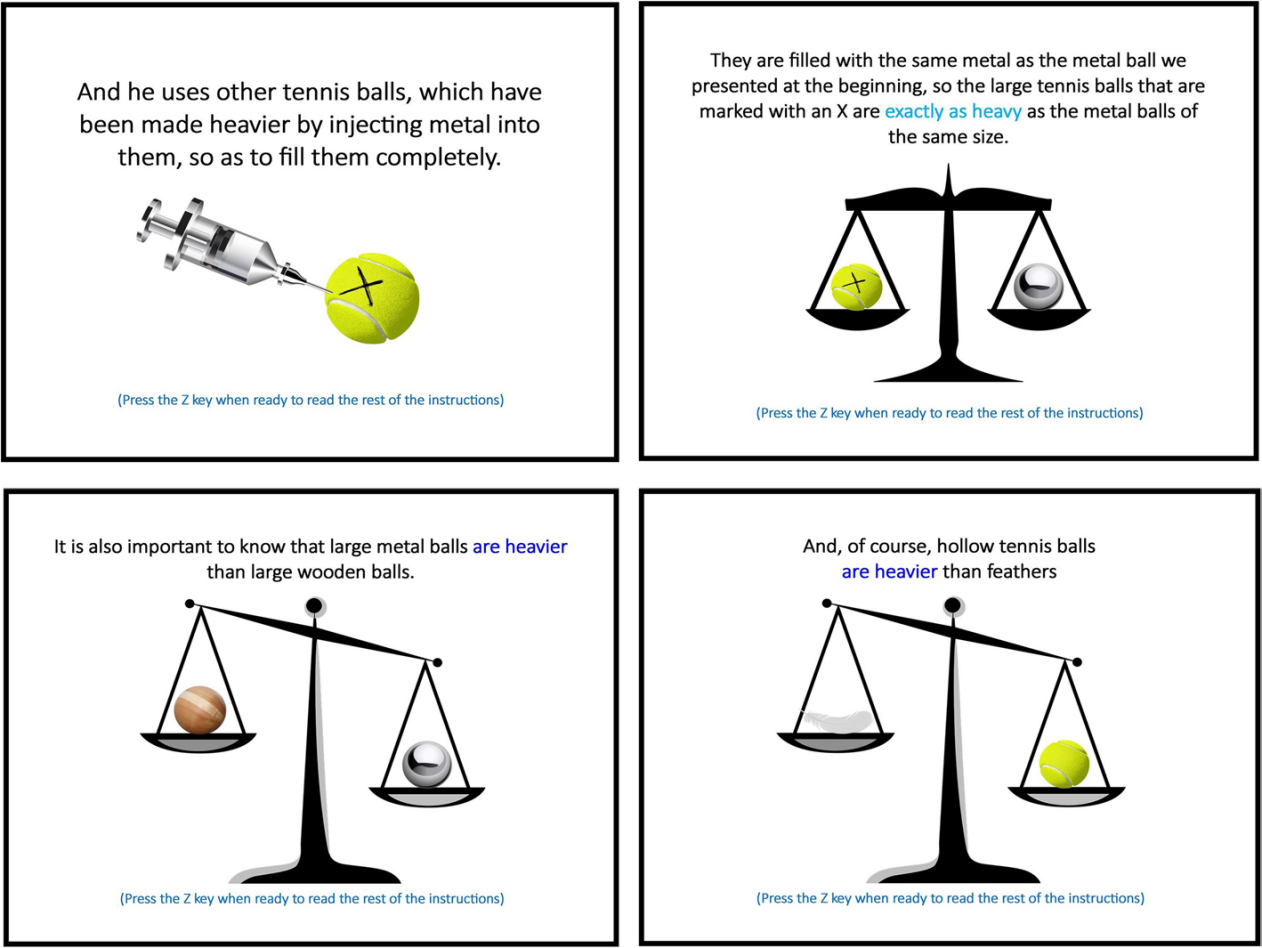
A few more slides from the instructions

Instructions then presented all possible objects to be tested, one by one, and provided short descriptions of each: small or large metal balls [SM and LM]; small or large (same sizes as metal) wooden balls [SW and LW]; normal (large) tennis balls [LT]; and metal-injected tennis balls [LTM (marked with an X)] and feathers [F]. All small objects were of the same size, as were all large objects (and feathers). Instructions then explained (through another series of slides) that weights are ordered as LM = LTM > LW≈SM≈LT > SW > F, using a Roman scale (Fig. 3).

**Fig. 4:**
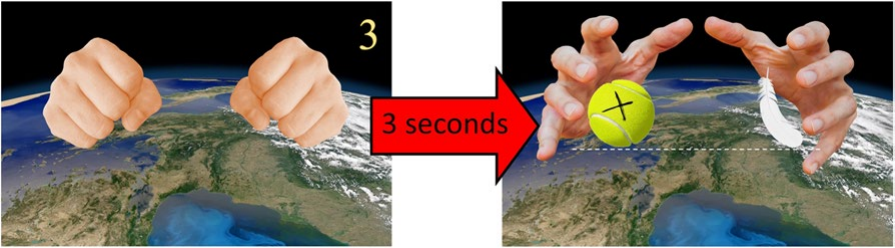
An example of a trial (this time on Earth): Trial 40

Participants then began. They had to produce 60 answers to each of our randomly ordered stimuli (to avoid sequencing effects), by choosing the “left”, “right” or “down” key when the hands opened and revealed the objects. First, for each trial, the hands were closed during three counted seconds, allowing the participant to assess whether the situation was contextualized on Earth or on the Moon (Fig. 4, left). Then, the hands opened (Fig. 4, right) and revealed the two objects. Participants had already been instructed to answer as fast as possible, but not at the expense of providing the best answers they could.

The entire set of stimuli used (*N* = 52) is available here, or through the corresponding author. A few of the stimuli (15, 16, 43 and 44) were repeated to ensure that enough data was gathered about each considered attractor (total of 60 trials). The description provides correct answers, as well as congruency for each stimulus, by conceptual attractor; that is, what a participant is expected to answer if he/she adheres to each considered attractor.

### Analysis

**Fig. 5:**
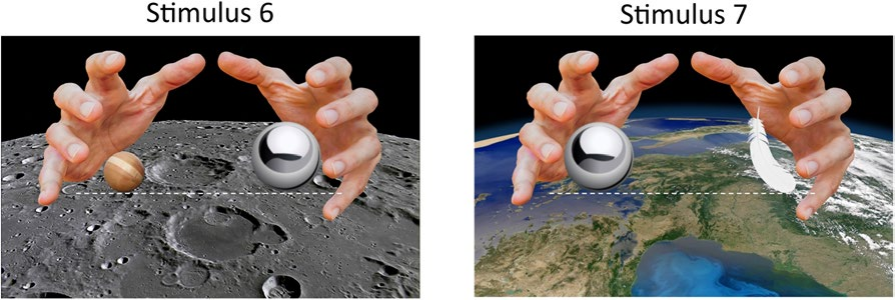
Stimuli 6 and 7

In order to provide answers to our research question, we will present, with regard to each hypothesis, the general inter-individual results regarding accuracies (means) for the entire set of data, by competence (schooling, and then relevant teaching experience). For the *prevalence* of each attractor, we will calculate the percentage of answers that correspond to the conceptual attractor’s congruency. For *interference*, we will calculate the difference in response times between correct answers that are incongruent with conceptual attractors and correct answers that are congruent with it (incongruent > congruent). By doing so, we will obtain a measure of the interference that a conceptual attractor may cause in the production of a correct answer, by comparison with the situation for which the interference cannot occur (congruent). For example, a participant who would correctly answer stimuli 6 and 7 (see Fig. 5; correct answers being “down” and “left” respectively) could experience a bit more trouble answering stimulus 6 because of a possible distracting (interfering) effect of MASS. Thus, the difference in response times between an incongruent (correct) answer to stimulus 6 and a congruent (correct) answer to stimulus 7 could be informative of the interference effect of MASS. Such interference scores cannot be obtained, however, for GALILEO and MASS-DRAG, because there is no correct-and-incongruent answer for those.

Then, for participants of all competency levels, we have performed Pearson’s correlations, as well as one-way ANOVAs, with Bonferroni corrections for all *post-hoc* tests in attempts to confirm hypotheses.

## Results and preliminary interpretations

### Descriptive statistics

We had data from 953 participants. We deployed recruitment efforts to obtain a minimum of 20 participants in each category of competence (schooling, and then relevant teaching experience). We succeeded, except for the extremes of the continuum: preschoolers and primary students [identified as PRIM] (*n* = 19); secondary [SEC] students (*n* = 388), secondary diplomas (*n* = 67); studied mechanics in 5^th^ secondary (247); in college [COL] (99); in university [UNI] (*n* = 35); having taught science in secondary levels (*n* = 36); having taught mechanics in 5^th^ secondary (*n* = 28); in college (*n* = 25); and in university (*n* = 9). We have refrained from integrating less populated extreme categories into their immediate neighbors because they sometimes showed interesting differences. However, we will remain very cautious in drawing conclusions about them.

It is possible that the diversity of recruitment methods (some through social media, others directly in the classroom, etc.) may have created unequal filtering effects, making our cross-category comparisons less valid. However, given the online nature of the task, it is very difficult to predict the direction of possible biases that could lead to unreliable data. We were aware of such a risk from the beginning; therefore, we made efforts to obtain a large number of participants in order to reduce such negative effects. The rather strong continuity of our results (see below) somewhat reassured us, but we remained cautious in our conclusions. To avoid the presence of missing information, we only considered data from fully completed tasks. Finally, we kept our task as short as possible (7 min) to avoid participant fatigue. However, it remains possible that younger participants may have experienced such a fatigue effect more. We suspect this because some of them reported feeling a bit bored at the end. However, no signs of fatigue were observed in participants older than PRIM.

### General accuracies

**Fig. 6:**
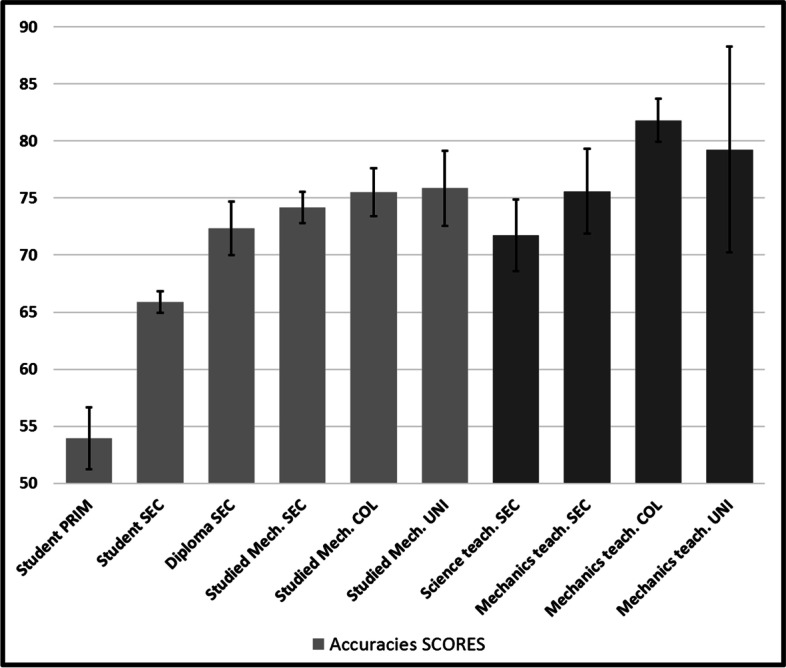
General accuracies

Figure 6 presents general accuracies (%), by competence (age and experience) with standard error bars. Darker bars indicate having taught science or mechanics (as in all following figures). There was a positive correlation between accuracies and experience (*r* = 0.312, *n* = 953,* p* < 0.001). A one-way ANOVA revealed statistically significant differences in general accuracy between at least two groups (F(9, 940) = 6.995, *p* < 0.001). Notice that lower-level performances may appear high (> 50%), possibly because of the nature of the task (three choices of answers every time). Even if *post-hoc* tests revealed significant differences of all four lowers levels with all higher ones (*p* <  = 0.037), no significant differences can be recorded between groups that have at least studied mechanics at the secondary levels. A general positive tendency can be observed all the way, except for secondary “general” science teachers’ group, which includes, for example, biology teachers, who have not taught mechanics, while nevertheless having necessarily studied it at the university level. We now turn our analysis to prevalence and interference assessments of all seven attractors, which the task’s format may allow revealing.

### Prevalence and interference

VOLUME- The analysis of adherence to this conceptual attractor revealed a positive correlation between the two variables (experience and attractor congruency) (r = 0.138, n = 953, *p* < 0.001), while ANOVA revealed few significant differences between groups (only for preschool and primary with higher levels), suggesting that this attractor was, at best, marginally mobilized by our participants, and possibly drowned by a more dynamic use of others. The analysis of interferences appears to support this conclusion, as no significant correlation (*p* = 0.078) was recorded, neither through ANOVA (*p* = 0.573). Mean interference scores are very weak for all groups (all under 100 ms). It is most likely that overcoming this attractor could be a challenge only for very young children and that not much after that, MASS swiftly becomes a much more important attractor.

**Fig. 7:**
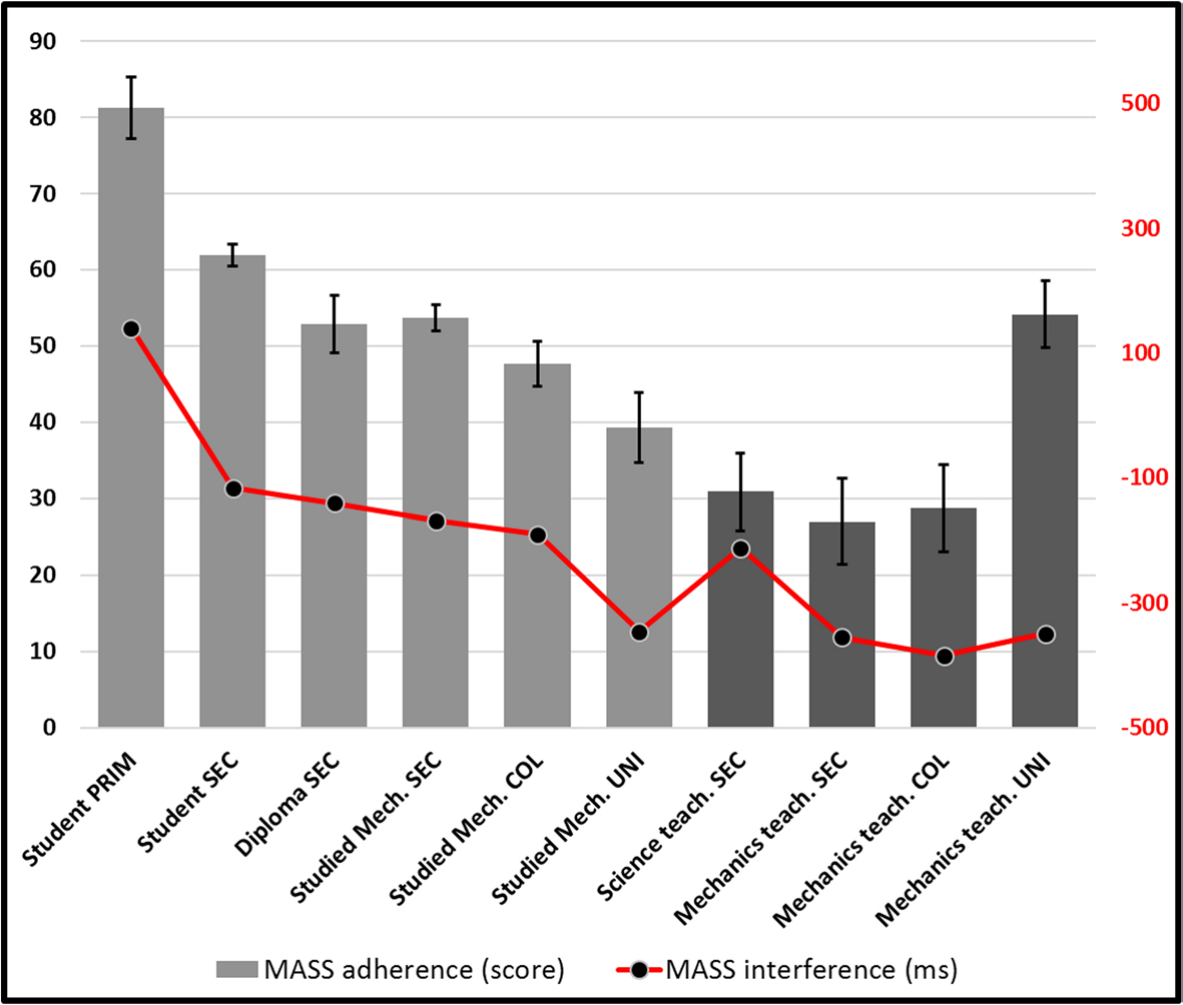
Adherence and interference for MASS

MASS- Fig. 7 shows adherence (grey bars- left scale (black)) as well as interference (red line- right scale [red]). Standard error bars were removed from interference points for clarity (in all following graphs). All standard deviations for response times are under 0.5 s, including in following graphs. One-way ANOVA revealed statistically significant differences in general adherence scores (F(9, 940) = 14.421, *p* < 0.001) as well as in interference (F(9, 819) = 2.306, *p* = 0.015). The graph thus shows a clear general declining tendency of adherence (*r* = -0.32, *n* = 953, *p* < 0.001), except for university teachers who appear to escape it (however not significantly, possibly because of the rather low number of participants in this group). *Post-hoc* tests reveal significant differences between most adherence score of lower-than-college students with higher experience groups, however higher groups not being distinguished (from one another) (*p* > 0.05).

These results suggests that physics training successfully appears to produce somewhat of a suspicion against the MASS-only “temptation”. However only university teachers appear to resist this “absolute” rejection of MASS (later, we will see that this will serve them). Interferences scores (in red) [distracting delays produced by the MASS attractor when answers are correct] appear to closely follow the tendency and support the decline hypothesis (*r* = -0.144, n = 832, *p* < 0.001). Post-hoc tests reveal nothing new. Except four our youngest group, all interference mean scores are negative, suggesting that this attractor in fact caused no hesitation, and rather supported (accelerated) the production of numerous correct answers, possibly a result of the structure of our task, in which many easier problems potentially mobilise this attractor.

**Fig. 8:**
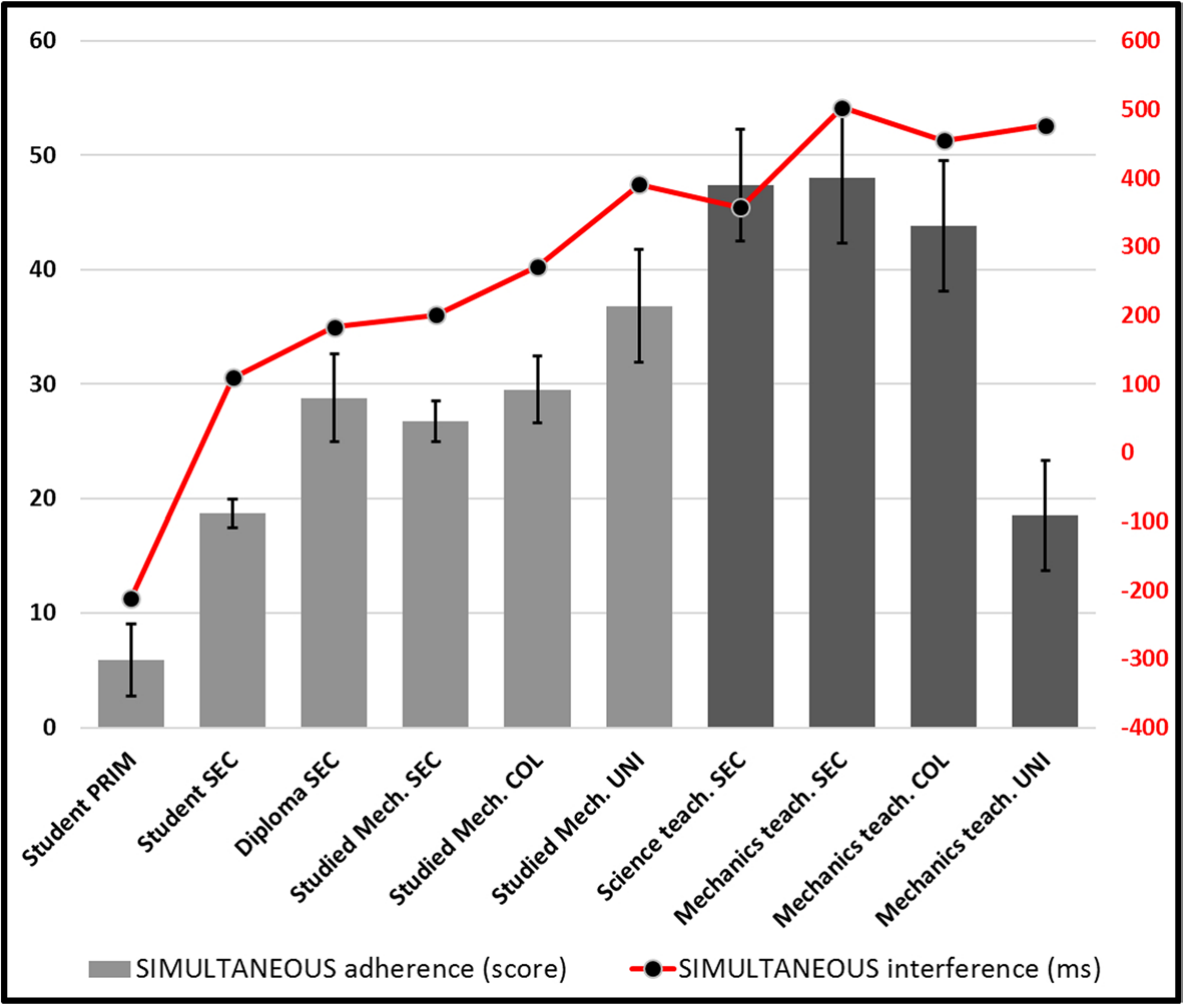
Adherence and interference for SIMULTANEOUS

SIMULTANEOUS- One-way ANOVA (Fig. 8) conducted on adherence (F(9, 940) = 11.210, *p* < 0.001) and interference (F(9, 925) = 8.192, *p* < 0.001) suggests a significant increasing tendency (*r* = 0.373, *n* = 953, *p* < 0.001) of competent participants to (unfortunately) mobilize this attractor (according to which objects will hit the ground at the same time, regardless of conditions) –Except, again, for university teachers. *Post-hoc* tests conducted on adherences reveal that most lower groups (5 lower categories) are significantly distinct from the all the one who studied mechanics at university (*p* < 0.05). Interference scores (rather high [most over 100 ms], and thus causing important hesitations that can reach as much as half a second for higher competence [age and experience] groups) appear to support the general increasing tendency (*r* = 0.264, *n* = 953, *p* < 0.001). However, this time, university professors appear to struggle against SIMULTANEOUS as much as all other experts. Indeed, *post-hoc* tests reveal that while most other groups can be distinguished (*p* < 0.05) from their immediate neighbour or the next, university professors’ interference scores cannot be distinguished (possibly because of their low population). These results suggest that science teachers from the secondary and college levels (taken together: *p* = 0.025) are surprisingly the strongest adherents to SIMULTANEOUS, and that they hypothetically could risk dragging along their students towards a rather oversimplified version of Galileo’s principle. We also believe that, combining this interpretation with the previous MASS attractor analysis, these teachers may actively promote SIMULTANEOUS, while at the same time actively discouraging MASS. This effect of training appears to be strong enough that all teachers (even from university, despite their low adherence) still must inhibit SIMULTANEOUS in specific cases in which they produce accurate answers –most likely in problems involving a dragging atmosphere.

**Fig. 9:**
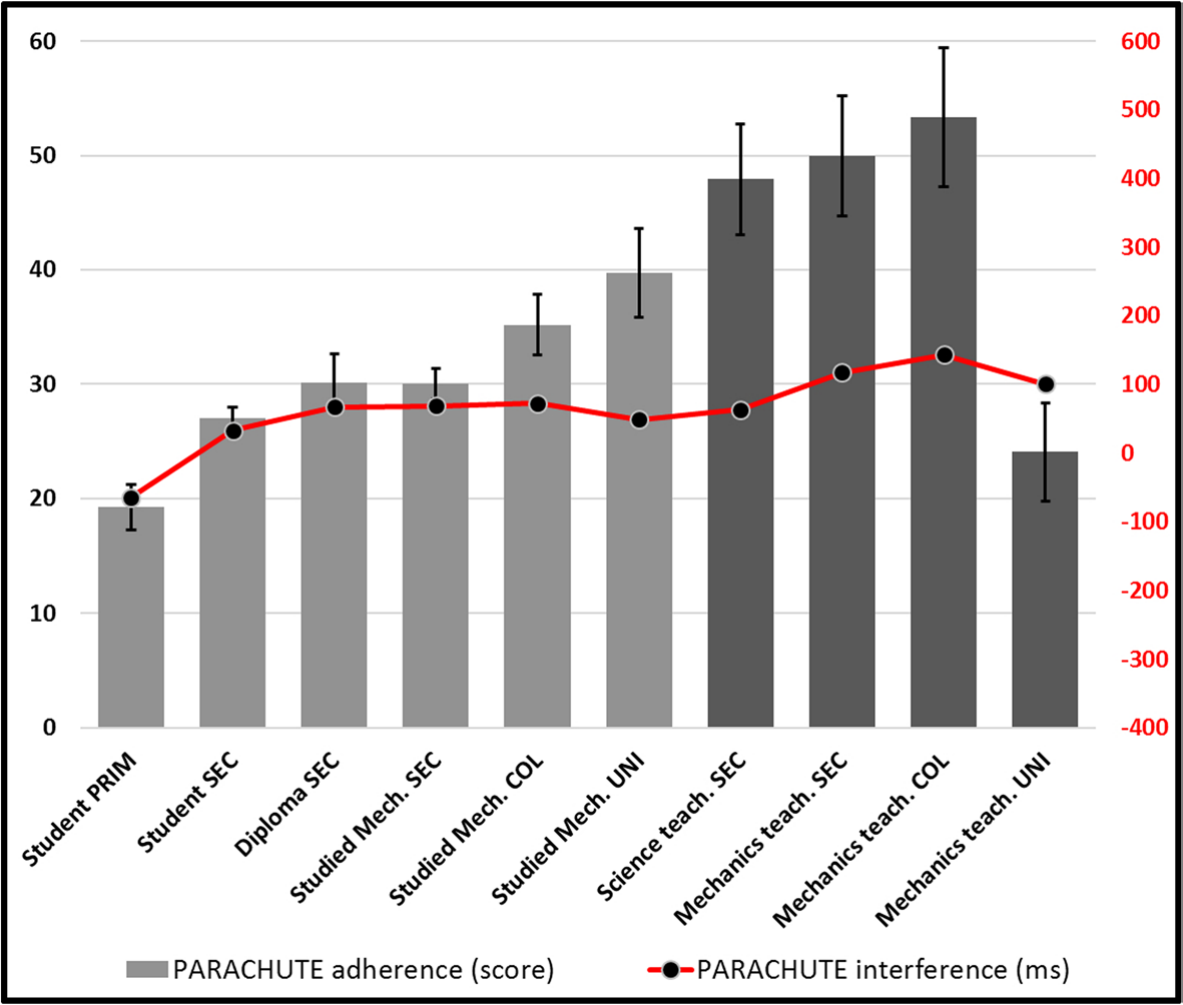
Adherence and interference for PARACHUTE

PARACHUTE- Fig. 9 presents scores for the *attractor* that larger objects (greater surface) will show *an increased tendency* to be slowed down, in all contexts. Adherence (F(9, 940) = 10.996, *p* < 0.001) show differences between groups, but interference (F(9, 940) = 1.366, *p* = 0.199) does not. The positive and rather low (generally under 100 ms) interference scores suggest that PARACHUTE is not an important obstacle in performance. However, the recorded general variation in adherence manifests in *post-hoc* analyses, while the four lower categories show significant differences with higher ones. Overall, the adherence to this attractor generally increases (*r* = 0.270, *n* = 953, *p* < 0.001), with university teachers again showing discrepancy; with significant differences with other mechanics teachers (*p* = 0.024). However, the very low scores of interferences (< 100 ms) and the weak variation (*r* = 0.106, *n* = 953, *p* < 0.001) suggest that participants do not struggle much with PARACHUTE when producing accurate answers. We believe this result to converge with the rather weak results we got for VOLUME, with which it is likely linked. Thus, neither VOLUME nor PARACHUTE appear to be among the strongest attractors our participants give in or resist to.

SMOOTH- ANOVA conducted for adherence (F(9, 940) = 10.996, *p* < 0.001) and for interference scores for this attractor (F(9, 938) = 10.996, *p* < 0.001) both show significant differences. However, since only a rather weak increasing tendency can be identified, we will make economy of presenting a figure. Noticeable is that interference scores are also weak (most between 100 ms and -100 ms), except for university teachers who hesitate a bit with it when they produce correct answers (hesitations of around 200 ms [n.s.]). They also show a non-significant correlation with experience (*r* = 0.053 *n* = 942, *p* < 0.106). We believe these general results to be in line with science education habits of generally discarding drag from the submitted problems, as well as explicitly choosing to ignore roughness (slickness, smoothness). Thus, students’ attentions could be more attracted to other considerations.

GALILEO- The analysis of adherence to this attractor is presented slightly differently from precedent ones: 1) because GALILEO is not a misconception, and it 2) its consideration is restricted to non-atmospheric problems. 3) It cannot include interferences scores (since all correct answers are aligned with it). Consequently, adherence scores are the same as accuracies (in all non-atmospheric problems).

**Fig. 10:**
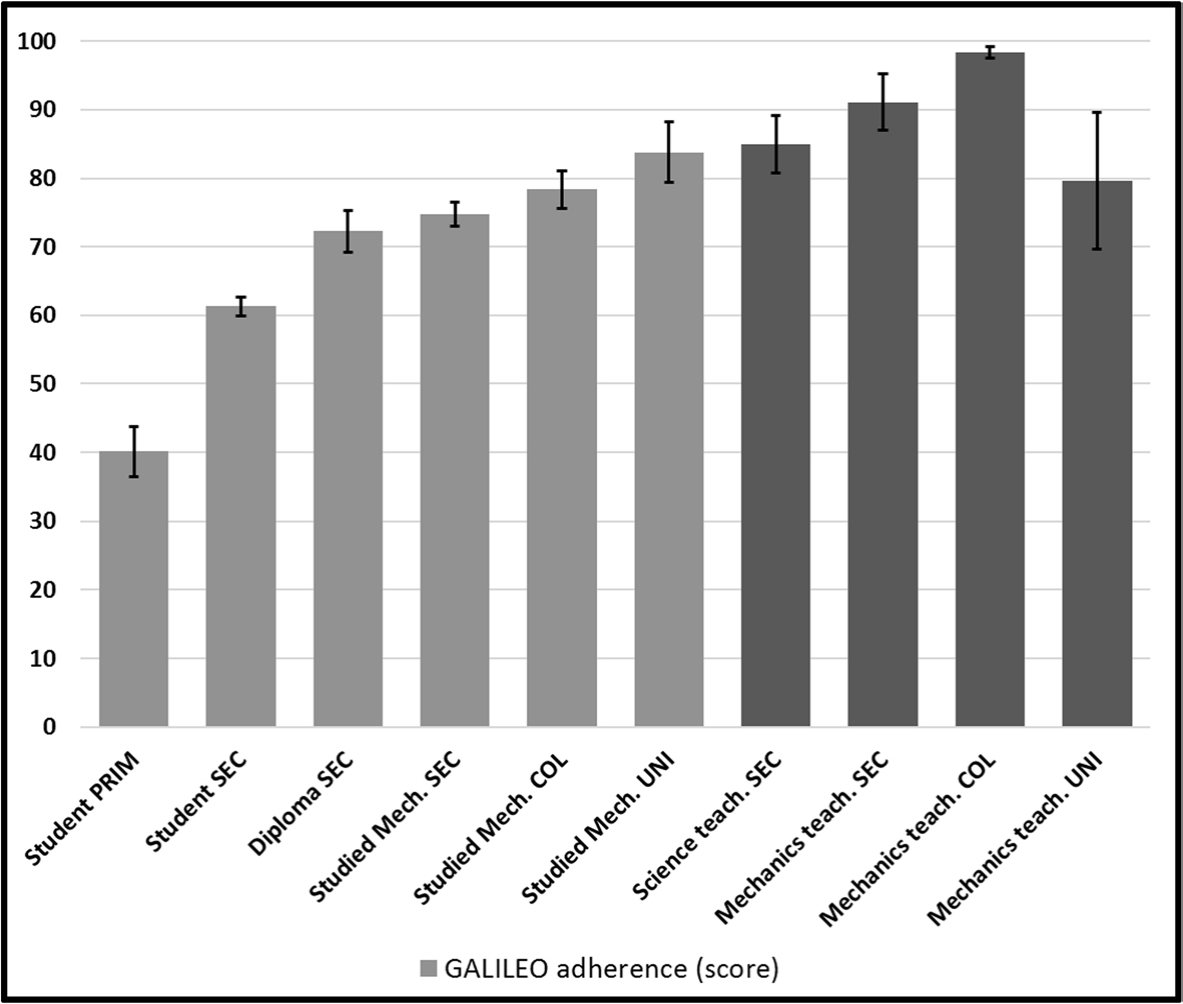
Adherence to GALILEO

Figure 10 shows the adherence scores (F(9, 940) = 16.0186, *p* < 0.001). Most *post-hoc* tests show that the five lower groups are each significantly different from all higher ones, and that the four higher ones are different from all lower (while not from each other). This result is encouraging, since scores generally increase with training (*r* = 0.344, *n* = 953, *p* < 0.001), and are getting farther from the 33% floor (that comes with a three-choice question). College mechanics teachers showed an almost perfect result, and all participants with university training showed higher than 85% scores.

MASS-DRAG- This analysis has been conducted similarly to the precedent one, except that it is restricted to atmospheric problems. The tested conceptual attractor well conformed to scientific knowledge and thus converged with age and experience (as GALILEO did).

**Fig. 11:**
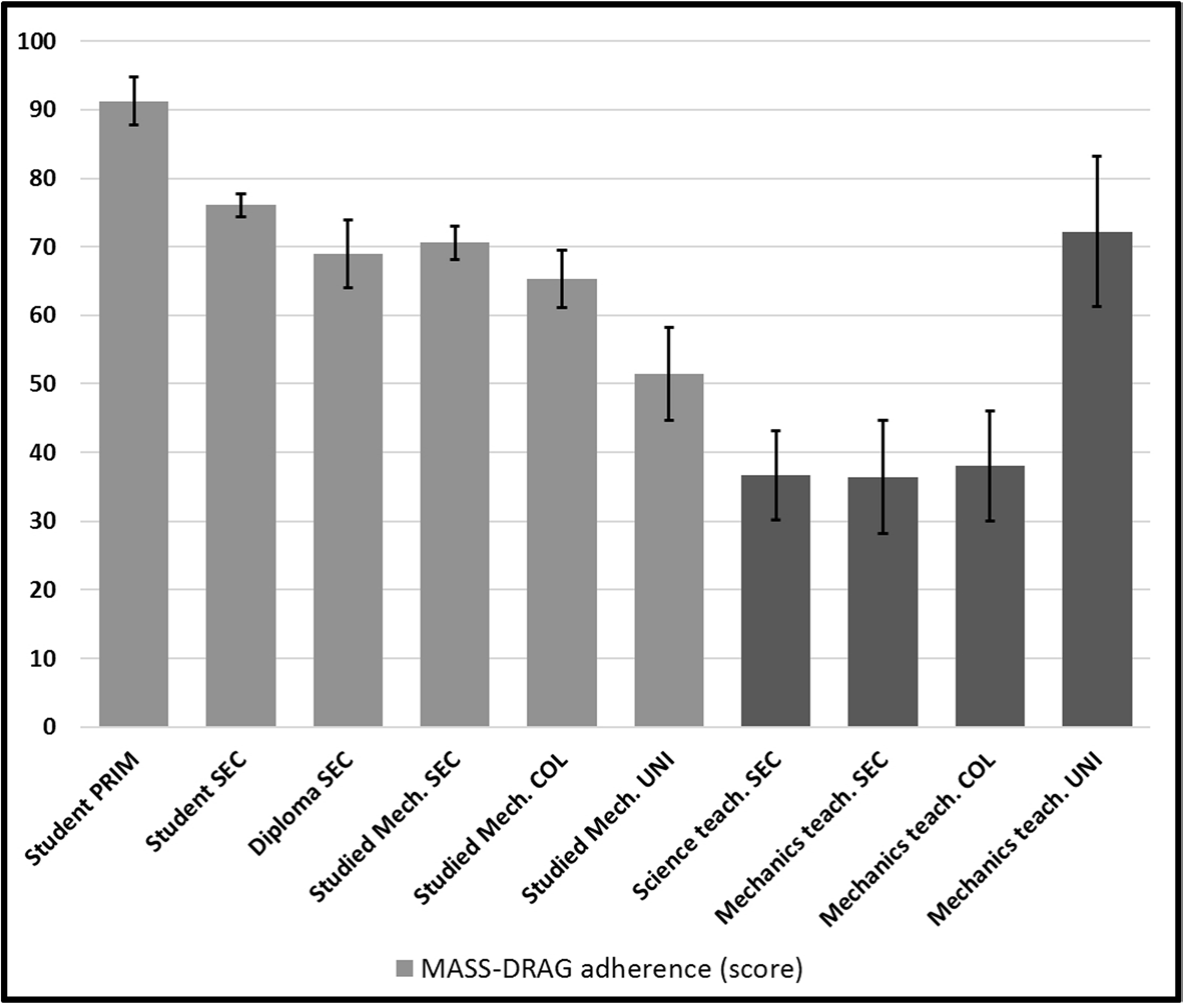
Adherence to MASS-DRAG

Results (Fig. 11) are a bit less encouraging and show a surprising and clear decreasing tendency (*r* = -267, *n* = 953, *p* < 0.001). One-way ANOVA revealed statistically significant differences (F(9, 940) = 10.736, *p* < 0.001). *Post-hoc* tests revealed that most non-teacher groups show differences (*p* < 0.05) with most of teacher groups, except university teachers, again. Sadly, secondary and college teachers show the lowest scores of all, near the 33% threshold of “null performance” that comes with our three-choice questions. Surprisingly, the best participants are simultaneously the less and most competent.

**Fig. 12:**
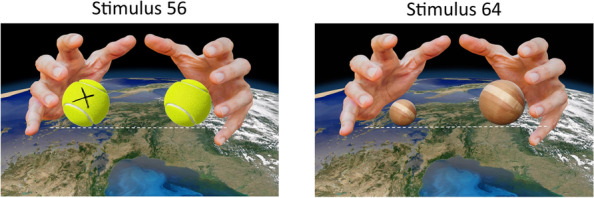
Stimuli 56 and 64

University professors appear to have managed to (very clearly) escape this trend, possibly because of a better understanding that comes with a thorough comprehension of the quantitative, or because of an extended exposition to, or deeper treatment of, falling bodies problems. For a task like the one we have administered, most people might resort to simpler principles or heuristics for resolution, especially in the context of time pressure. It is thus possible that a better theoretical familiarity with the quantitative treatment of the crucial variables was necessary to provide a scientifically based resolution, for stimuli like No.56 (large tennis ball injected with metal + large tennis ball [correct = left]), or No. 64 (small wooden ball + large wooden ball [correct = right]) (Fig. 12).

We believe the recorded unfortunate decline of MASS-DRAG to be attributable to a competition with SIMULTANEOUS, which conflicts with it in atmospheric contexts, suggesting that low adherence scores result from a defective use of GALILEO in atmospheric settings.

## Discussion

### General highlights

Not surprisingly, our data support the hypothesis that conceptual understanding of falling body problems improves with age and experience (Fig. 6). Surprisingly, most conceptual gains appear to occur before secondary school, in the absence of any physics training, suggesting that this improvement may be developmental, or that direct experience rapidly corrects certain misconceptions, such as VOLUME. Paradoxically, the improvement slows down when physics training begins. Thus, our hypotheses are only partly confirmed.

A more thorough analysis, including the prevalence (correct answers) and interference (delays or hesitations caused by irrelevant distractors) scores obtained for each conceptual attractor, allows us to go deeper in our interpretation. First, it shows that MASS (Fig. 7) is initially prevalent, as has been noted before (Gunstone & Watts, 1985; Sequeira & Leite, 1991; Whitaker, 1983), but regularly decreases along our competence (age and experience) continuum, suggesting that not only direct interactions with the world, but also training contribute to reducing its perceived cognitive utility and its interference power.

However, it is possible that training efforts may be pushing a bit too hard to promote its rejection, since interference is negative, suggesting that it ultimately contributes to the production of incorrect answers. More than everybody else, high school and college physics teachers appear to be strongly opposed to MASS, except university physics teachers, who also show the best overall scores. It is not unreasonable to suggest here that physics teaching at lower levels may sometimes commit the sin of generalizing an indiscriminate rejection of mass as a relevant variable. To our knowledge, these results are the first to document this shortcoming as convincingly.

This suggestion is reinforced by the recorded increase of GALILEO (Fig. 10) and of SIMULTANEOUS (Fig. 8) to which MASS is probably systematically and explicitly opposed in physics courses. The interference that SIMULTANEOUS generates is also very strong after the first physics course (> 300 ms) and maintains even when the strongest physicists of our cohort (university teachers) produce correct answers (> 500 ms) for the most difficult stimuli of our task. Champagne et al. (1980) had already hypothesized the importance of this misconception. SIMULTANEOUS thus unfortunately appears to be a strong product of teaching efforts, as Cahyadi and Butler (2004) and Song et al. (1996) have suggested, possibly as much as MASS may have been too recklessly rejected, even in atmospheric settings, much as Lehavi and Galili (2009) have argued. MASS-DRAG scores (Fig. 11), which unfortunately decline throughout our “age and experience” continuum, also reinforce this conclusion. Only university physics teachers mobilize MASS-DRAG enough to avoid this pitfall. Ironically, they are catching up with our less competent participants and are the only ones who are going beyond the misconceptions that prevail at the secondary and college levels.

This result is a bit worrying since these teachers are precisely the ones who teach physics to children. Declines that begin at the secondary level suggest that all involved in school business, learners and teachers alike, cultivate this misapprehension of atmospheric falling objects problems. It is somewhat paradoxical, however, that scores on the PARACHUTE attractor (Fig. 9) also generally increase with experience, with physics teachers being the strongest adherents. We can hypothesize that they mostly resort to this misconception only in very special cases, for example to explain why the fall of very oddly shaped objects, like feathers, appears longer in the atmosphere, mostly in cases of falls from low heights. The rather low interference (< 120 ms) by PARACHUTE however suggests that participants do not let it get a lot in their way in most of their reflections.

The general decrease of MASS-DRAG, the desired attractor, is rather bad news and supports the hypothesis that many teachers have difficulty simultaneously integrating all relevant variables in falling bodies problems, especially in atmospheric settings. Figure 11 teaches us that their adherence to this heuristic is in fact the lowest (except for university professors), nearing the floor of 33% that comes with three-choices items.

In their defense, however, it must be remembered that the participants were pushed to answer quickly and without the opportunity to develop their reasoning in writing on a piece of paper or on the blackboard. It remains possible that high school and college teachers would have produced better answers if they had been given a pencil, a piece of paper, and all the time they needed. Physics and falling body problems are known to be counterintuitive. It is possible, therefore, that asking participants to produce a series of correct answers repeatedly and quickly in such a context might be considered somewhat unfair. However, the university professors were rushed just as much as the other participants, and the response times of their (more often correct) answers were comparable. It seems a bit disturbing, then, that except for university professors, physics teachers-and indeed all science teachers-may very well be the source of certain misconceptions in their students, or of the contextually inappropriate use of some conceptual attractors. Could it be that the teachers performing their duties are so professionally isolated from one another that sufficient mutual regulation cannot be expected? Do the documents they use (textbooks, handbooks, and e-resources) lead them to make mistakes? Could we be in the presence of *supermisconceptions* that even our most competent practitioners cannot deal with?

At the end of this analysis, we can also see that the conceptual attractors of VOLUME and SMOOTH(-ness) did not play an important role in the decision processes of our participants. While the adherence and interference of VOLUME was recorded only by the very youngest participants, SMOOTH was recorded only by the oldest. Nor did PARACHUTE produce much interference. This lack of result (in contrast to the high slopes and interference scores produced by MASS and SIMULTANEOUS) may support the hypothesis that considerations related to drag are not sufficiently addressed in training and are too often (methodologically?) discarded. Indeed, drag should never be neglected in atmospheric problems, and it is directly related to the volume, shape, and texture of falling objects. Our students simply did not appreciate that these played an important role in the solution.

### Contribution to practice

From our data, it is possible to suggest that high school and college physics teachers might benefit from varying the cases they invoke when teaching falling bodies, rather than simply focusing on too few idealized examples that validate a simple effect of one variable on another. Free fall through a dragging atmosphere is a complex, multivariate problem, and it is very difficult to find realistic and qualitatively *formulatable* cases in which only one independent variable is changed. The example of the “large wooden ball + small wooden ball” is typical; the large ball indeed also has a larger mass, but also a larger cross-section. Considering the "pebble + feather" problem, let's just say that it can hardly be an effective and sufficient illustration of the PARACHUTE effect due to inevitable confounding variables. Nevertheless, we believe, like Vicovaro (2014), that the consideration of pairs of objects is a profitable strategy, provided that many of these pairs (or trios or quartets…) are simultaneously considered, treated, and explicitly and systematically compared, most likely in light of all relevant variables/vectors, and with a thorough analysis of their simultaneous integration.

Physics teachers also likely should be cautious when teaching Galileo’s principle. It is indeed valid in a vacuum but cannot be extrapolated in its simplest form to atmospheric contexts. The same goes for the important value “9.8 m/s^2^”. Often presented as the “gravitational acceleration at the surface of the Earth,^1^” it might benefit from being presented as the “gravitational acceleration at the surface of the Earth *that we’d observe if there was no deceleration force (i.e., producing negative, upward acceleration) due to the atmosphere*”. The fact that 9.8 m/s^2^ is presented as a *constant* could also cause certain problems; it might be understood as falls inevitably happening at this precise rate, or even as the *cause* of movement. But accelerations are never causes; they are effects of forces. Thus, one must always consider all relevant forces to infer the eventual net acceleration of a hard object. A side effect of understanding g = 9.8 m/s2 as a cause, or as an inevitability, might also be the conclusion that if both falling objects are subjected to the same friction (same surface texture, shape, and volume), then their rates of fall will necessarily be equivalent. Most likely, such an inference could be a major cause of the observed failures to correctly answer the “empty tennis ball + metal filled tennis ball” problem. Some physics teachers could then possibly benefit from integrating careful analyses of cases such as “small pebble + big rock” and “beach ball filled with air + beach ball filled with water,” and adding such problems to their arsenal of examples. Since these problems are counterintuitive and thus difficult to treat without rigorous mathematical analysis (i.e., vectorial sum of forces), teachers could encourage students to be vigilant and aware, to exercise deeper reflection, and to overcome the SIMULTANEOUS temptation. Some of them could also choose to promote the use of an attentive mathematical treatment of falling bodies problems in a way that could ultimately lead to a better and more systematic resistance (inhibition) to certain indiscriminate uses of some attractors, such as SIMULTANEOUS and PARACHUTE (but maybe not to MASS, which appears to be already challenged enough).

### Contribution to research

We believe that our research design could be useful to other researchers in the field of science education who are interested in conceptions and misconceptions. We believe that it can provide an interesting analysis grid that can lead to reasonable assessments or recommendations. The simplicity of the task and of the prompts (left/right/down) also allows for interesting and valid comparisons between participants with a wide range of competencies, thus also allowing for broad transversal analyses involving many attractors simultaneously, in a Siegler-inspired way (Fig. 1). Thus, our study seems (to us) to be a reasonable contribution, since we have not found any other lifelong analysis of conceptual development in the literature, let alone one using a single task. However, the method cannot be extended to every conceivable conception/misconception, since not all of them can be reduced to mere static stimuli with so few possible answers. Our task may provide access to deep and intuitive/phenomenological attachments, but not to the time-consuming reasoning that physics so often requires. We believe that it may then remain blind to situations in which physics is at its best: analytical, methodical, and self-aware reflection.

We also believe that our design provides an interesting account of conceptual change problems through its framing within a prevalence/pluralist (Potvin, 2022) perspective. The interferences we recorded appear to converge with other results, obtained with accuracies, but also allow additional interpretations.

## Conclusion

This research allowed us to explore the development of an understanding of falling bodies that supports, adequately or not, the resolution of certain physics problems both in an atmosphere and in a vacuum. Using the prevalence framework, we have interpreted data extracted from the use of a cognitive task that recorded answers and latencies. Our conclusion led us to believe that while some misconceptions are quickly suppressed with age or formal instruction, others may be the result of schooling. High school and college science (physics) teachers seem to widely hold the belief that differently weighted objects will necessarily fall at exactly the same rate in atmospheric contexts, especially if their characteristics suggest that the friction (with the fluid through which they fall) will be equivalent for both objects. This belief is likely to result in teaching efforts that explicitly oppose the “naive” notion that mass is the essential key to solving falling body problems, to the no less naive attractor that mass never is; that falling objects will always accelerate at the same rate (9.8 m/s2), regardless of mass, or provided that friction is equivalent for all objects considered. Data from student participants seem to confirm this problem. Moreover, only university physics professors seem to avoid this pitfall.

We hope that our results will make physics-mechanics teachers aware of their possible adherence to, or misuse of, certain conceptual/misconceptual attractors. We also hope that researchers will see in our results yet another indication that didactic problems such as falling bodies should not be viewed as a succession of monolithic, isolated ideas to which adherence is exclusive and sequential, but rather as an evolving and organically woven web of conceptual resources that can nonetheless inform us about where to focus the subsequent innovation efforts.

## Data Availability

The datasets used and/or analysed during the current study are available from the corresponding author on reasonable request.

## Footnotes

1 Often, the second part of the message, about the height at which the acceleration is valid and usable, is not even specified.

## References

[CR1] Allaire-Duquette G, Brault Foisy L-M, Potvin P, Riopel M, Larose M, Masson S (2021). An fMRI study of scientists with a Ph.D. in physics confronted with naive ideas in science. npj. Science Learning.

[CR2] Arons AB, Redish EF (1997). Teaching introductory physics.

[CR3] Bayraktar S (2009). Misconceptions of Turkish Pre-Service Teachers about Force and Motion. International Journal of Science and Mathematics Education.

[CR4] Bélanger, M., & Potvin, P. (2022). Bridging pluralisms. In M. Bélanger, P. Potvin, S. Horst, A. Shtulman, & E. F. Mortimer (Eds.), Multidisciplinary perspectives on representational pluralism in human cognition. tracing points of convergence in psychology, science education and philosophy of science. Routledge. 10.4324/9781003189930-3

[CR5] Brault Foisy L-M, Potvin P, Riopel M, Masson S (2015). Is inhibition involved in overcoming a common physics misconception in mechanics?. Trends in Neuroscience and Education.

[CR6] Brown DE (1993). Refocusing Core Intuitions : A Concretizing Role for Analogy in Conceptual Change. Journal of Research in Science Teaching.

[CR7] Brown DE, Hammer D, Vosniadou S (2008). Conceptual change in physics. International handbook of research on conceptual change.

[CR8] Cahyadi MV, Butler PH (2004). Undergraduate students' understanding of falling bodies in idealized and real-world situations. Journal of Research in Science Teaching.

[CR9] Champagne AB, Klopfer LE, Anderson JH (1980). Factors influencing the learning of classical mechanics. American Journal of Physics.

[CR10] Chi M, Giere RN (1992). Conceptual change in and across ontological categories: Exmaples for learning and discovery in science. Cognitive models of science.

[CR11] Chinn CA, Malhotra BA (2002). Children's responses to anomalous scientific data: How is conceptual change impeded?. Journal of Educational Psychology.

[CR12] diSessa AA (1993). Toward an Epistemology of Physics. Cognition and Instruction.

[CR13] diSessa AA, Sawyer RK (2006). A History of Conceptual Change Research: Threads and Fault Lines. The Cambridge handbook of the learning sciences.

[CR14] Ferreira A, Seyffert AS, Lemmer M (2017). Developing a graphical tool for students to understand air resistance and free fall: when heavier objects do fall faster. Physics Education.

[CR15] Ganea, P. A., Larsen, N. E., & Venkadasalam, V. P. (2020). The role of alternative theories and anomalous evidence in children’s scientific belief revision. *Child development*, *n/a*(n/a). 10.1111/cdev.13481 10.1111/cdev.13481 33378117

[CR16] Gunstone, R., & Watts, M. (1985). Force and motion. *Children’s ideas in science*, 85–104

[CR17] Hake RR (1998). Interactive-engagement versus traditional methods: A six-thousand-student survey of mechanics test data for introductory physics courses. American Journal of Physics.

[CR18] Halloun IA, Hestenes D (1985). Common sense concepts about motion. American Journal of Physics.

[CR19] Hast M, Howe C (2012). Understanding the beliefs informing children’s commonsense theories of motion: The role of everyday object variables in dynamic event predictions. Research in Science & Technological Education.

[CR20] Kaiser MK, Proffitt DR, McCloskey M (1985). The development of beliefs about falling objects. Perception & Psychophysics.

[CR21] Kavanagh, C., & Sneider, C. (2007). Learning about gravity I. free fall: a guide for teachers and curriculum developers. *Astronomy Education Review,**5*(2), 21–52

[CR22] Kelly GJ, Green J, Guzetti B, Hynd C (1998). The social nature of knowing: Toward a sociocultural perspective on conceptual change and knowledge construction. Perspectives on conceptual change: Multiple ways to understanding, knowing and learning in a complex world.

[CR23] Lehavi Y, Galili I (2009). The status of Galileo’s law of free-fall and its implications for physics education. American Journal of Physics.

[CR24] Mason L (2007). Introduction: Bridging the Cognitive and Sociocultural Approaches in Research on Conceptual Change: Is it Feasible?. Educational Psychologist.

[CR25] Masson S, Potvin P, Riopel M, Brault Foisy L-M (2014). Differences in Brain Activation Between Novices and Experts in Science During a Task Involving a Common Misconception in Electricity. Mind, Brain, and Education.

[CR26] Nussbaum J, Novick S (1982). Alternative Frameworks, Conceptual Conflict and Accommodation : Toward a Principled Teaching Strategy. Instructional Science.

[CR27] Perret-Clermont A-N (1996). La construction de l'intelligence dans l'interaction sociale [The construction of intelligence in social inteaction].

[CR28] Posner G, Strike K, Hewson PW, Gertzog W (1982). Accomodation of a Scientific Conception : Toward a Theory of Conceptual Change. Science Education.

[CR29] Potvin P, Cyr G (2017). Toward a durable prevalence of scientific conceptions: Tracking the effects of two interfering misconceptions about buoyancy from preschoolers to science teachers. Journal of Research in Science Teaching.

[CR30] Potvin P, Masson S, Lafortune S, Cyr G (2015). Persistence of the intuitive conception that heavier objects sink more: A reaction time study with different levels of interference. International Journal of Science and Mathematics Education.

[CR31] Potvin, P., Malenfant-Robichaud, G., Cormier, C., & Masson, S. (2020). Coexistence of misconceptions and scientific conceptions in chemistry professors: a mental chronometry and fMRI Study [Original Research]. Frontiers in Education, 5(180). 10.3389/feduc.2020.542458

[CR32] Potvin, P. (2022). From conceptual change to conceptual pluralism. What the acknowledgement of representational plurality could mean for science teaching. In M. Bélanger, P. Potvin, S. Horst, A. Shtulman, & E. F. Mortimer (Eds.), Multidisciplinary Perspectives on Representational Pluralism in Human Cognition. Tracing points of convergence in psychology, science education and philosophy of science. Routledge. 10.4324/9781003189930-10

[CR33] Sherin B (2006). Common sense clarified: The role of intuitive knowledge in physics problem solving. Journal of Research in Science Teaching.

[CR34] Shtulman A, Valcarcel J (2012). Scientific knowledge suppresses but does not supplant earlier intuitions. Cognition.

[CR35] Sequeira, M., & Leite, L. (1991). Alternative conceptions and history of science in physics teacher education. *Science Education,**75*(1), 45–56

[CR36] Skelling-Desmeules, Y., Brault Foisy, L.-M., Potvin, P., Lapierre, H. G., Ahr, E., Léger, P.-M., Masson, S., & Charland, P. (2021). Persistence of the “Moving Things Are Alive” Heuristic into Adulthood: Evidence from EEG. *CBE—Life Sciences Education*, *20*(3), ar45. 10.1187/cbe.19-11-0244 10.1187/cbe.19-11-0244 PMC8715811 34388004

[CR37] Siegler RS (1996). Emerging minds: The process of change in children’s thinking.

[CR38] Song J-W, Jang K, Pak S-J (1996). Students' conceptions and the historical change of the concept: Free-fall motion. Journal of the Korean Association for Science Education.

[CR39] Stavy R, Tsamir P, Tirosh D, Lin FL, McRobbie C (2006). Are intuitive rules universal?. International Journal of Science and Mathematics Education.

[CR40] Syuhendri, S., Andriani, N., & Taufiq, T. (2019). Preliminary development of conceptual change texts regarding misconceptions on basic laws of dynamics. Journal of Physics: Conference Series, 1166 (012013). 10.1088/1742-6596/1166/1/012013

[CR41] van der Ven SHG, Boom J, Kroesbergen EH, Leseman PPM (2012). Microgenetic patterns of children’s multiplication learning: Confirming the overlapping waves model by latent growth modeling. Journal of Experimental Child Psychology.

[CR42] Van Hise YA (1988). Student misconceptions in mechanics: An international problem?. The Physics Teacher.

[CR43] Vicovaro M (2014). Intuitive physics of free fall: An information integration approach to the mass-speed belief. Psicológica.

[CR44] Vosniadou S (1994). Capturing and modeling the process of conceptual change. Learning and Instruction.

[CR45] Wang Z, Wang L, Wright JD (2015). Cognitive development: Child education. International encyclopedia of the social & behavioral sciences.

[CR46] Whitaker RJ (1983). Aristotle is not dead: Student understanding of trajectory motion. American Journal of Physics.

[CR47] Zhu Y, Zhang L, Leng Y, Pang R, Wang X (2019). Event-Related Potential Evidence for Persistence of an Intuitive Misconception About Electricity. Mind, Brain, and Education.

